# Validated instruments used to measure attitudes of healthcare students and professionals towards patients with physical disability: a systematic review

**DOI:** 10.1186/1743-0003-7-55

**Published:** 2010-11-09

**Authors:** Wai Yim Lam, Sameer K Gunukula, Denise McGuigan, New Isaiah, Andrew B Symons, Elie A Akl

**Affiliations:** 1 School of Medicine and Biomedical Sciences, State University of New York at Buffalo, NY, USA; 2 Department of Family Medicine, State University of New York at Buffalo, NY, USA; 3 Department of Medicine, State University of New York at Buffalo, NY, USA; 4 Department of Clinical Epidemiology and Biostatistics, McMaster University, Hamilton Canada

## Abstract

**Background:**

Instruments to detect changes in attitudes towards people with disabilities are important for evaluation of training programs and for research. While we were interested in instruments specific for medical students, we aimed to systematically review the medical literature for validated survey instruments used to measure attitudes of healthcare students and professionals towards patients with physical disability.

**Methods:**

We electronically searched Medline, EMBASE, PsycINFO, Health and Psychosocial Instruments. We included papers reporting on the development and/or validation of survey instruments to measure attitudes of healthcare students and professionals towards patients with physical disability. We excluded papers in which the attitudes were not measured in a provider-patient context. Two reviewers carried out titles and abstracts screening, full texts screening, and data abstraction in a duplicate and independent manner using standardized and pilot tested forms.

**Results:**

We identified seven validated survey instruments used for healthcare students and professionals. These instruments were originally developed for the following target populations: general population (n = 4); dental students (n = 1); nursing students (n = 1); and rehabilitation professionals (n = 1). The types of validity reported for these instruments were content validity (n = 3), criterion-related validity (n = 1), construct validity (n = 2), face validity (n = 1), discriminant validity (n = 1), and responsiveness (n = 1). The most widely validated and used tool (ATDP) was developed in the late 1960s while the most recent instrument was developed in the early 1990s.

**Conclusion:**

Of the seven identified validated instruments, less than half were specifically designed for healthcare students and professionals and none for medical students. There is a need to develop and validate a contemporary instrument specifically for medical students.

## Background

Three main issues have been identified in addressing the problem of health care providers and their approach to people with disabilities: lack of disability-specific knowledge; discomfort with working with people with disabilities; and attitudes and misperceptions about disability. People with disabilities have cited negative attitudes and behaviors of health care providers as the most formidable barriers to accessing health care services[1-5].

Negative attitudes held by health care providers about patients with disabilities may affect care that the patient receives. Although these attitudes and misconceptions are usually not overtly hostile, they may result in patients with disabilities not receiving appropriate treatment or not receiving indicated preventive care[2,6-8]. For example, physicians might defer a pelvic exam in a patient with a disability due to the misconception that these patients are generally not sexually active. The assumption that a patient with a disability has a baseline quality of life which is low may lead the physician to defer aggressive treatment of acute problems[3,9]. Adverse outcomes may be compounded and services available to patients may be limited if these subtle attitudes unduly affect the physician's judgment and actions. [5]

Until recently, disability has not been appropriately addressed in medical school curricula [2,6,10-14]. Larson McNeal, et al. surveyed practicing physicians in California and found that 22% had not received training in disabilities and acknowledged a need for such training[15]. A recent survey of dental and medical educoator and students in the United States identified a need for increased didactic and clinical preparation in the care of individuals with disabilities [16].

Several medical schools are currently involved in implementing curricula to improve students' knowledge, attitude and skills regarding caring for patients with disabilities. There are also calls on many levels to expand efforts in this area[14,17-22]. If these curricula are to be robust, there is a need for evaluation strategies - including validated instruments - to evaluate their effectiveness and guide their development.

While we were interested in instruments for medical students we aimed to identify instruments for healthcare students and professionals in general as they could be potentially useful for our aim. Also given the nature and characteristics of attitudes might vary by type of disability, we decided to focus on physical disabilities. Thus, our objective was to systematically review the medical literature for validated survey instruments used to measure attitudes of healthcare students and professionals towards patients with physical disability.

## Methods

### Eligibility criteria

We included papers reporting on the development and/or validation of survey instruments to measure attitudes of healthcare students and professionals towards patients with physical disability. We included instruments that measured exclusively physical disability as well as instruments that measured a range of disabilities that included physical disability. We used the following definition of attitude: a learned disposition directing feelings, thoughts and actions [4,23,24]. Validity is defined as the extent to which an instrument measures what it is supposed to measure. We opted not to define minimum eligibility criteria for validity while being inclusive and rigorously assess all aspects of instrument validity, including face validity, content validity, reliability, discriminant validity, and responsiveness. We included instruments developed for non-healthcare populations and used with healthcare students and professionals. We considered studies of any type, including qualitative studies, if used to validate a quantitative instrument. We excluded qualitative studies of the attitudes towards patients with physical disability that were not part of the validation of process of a quantitative instrument. We excluded papers in which the attitude was not measured in a provider-patient context, e.g. we excluded studies assessing the attitude of nurses toward co-workers with disability. We also excluded non-English reports.

### Search strategy

We conducted a comprehensive search for studies relating to attitude toward physically disabled individuals in June 2009. We searched the following electronic databases from their dates of inception: Medline (1950-present), EMBASE (1980-present), PsycINFO (1967-present), Health and Psychosocial Instruments (1985-present). Additional file 1 provides the electronic search strategies. Two medical librarians reviewed the search strategy to ensure its validity. Additionally, we screened the citation lists of included and relevant papers for potentially eligible studies.

### Selection process

In a first step, two reviewers screened for potential eligibility the title and abstract of identified citations in a duplicate and independent manner. We retrieved the full texts of citations judged as potentially eligible by at least one reviewer. In a second step, two reviewers screened for eligibility the retrieved full texts using a standardized and pilot tested screening form in a duplicate and independent manner. The two reviewers resolved their disagreements by discussion or by consulting a third reviewer. The team conducted calibration exercises for each of these steps to ensure consistency and validity of the process. The calibration exercises consisted of each team member screening several full texts and determining their eligibility to ensure that the entire team understood and followed the same screening criteria.

### Data abstraction

Two reviewers used a standardized and pilot tested form to abstract data from each eligible study in a duplicate and independent manner. They resolved their disagreements by discussion or by consulting a third reviewer. We extracted data relating to:

1. The name of the instrument and the concept being measured. While the main concept of interest was attitudes (which is in the affective domain), we also included instruments measuring perceptions (which is in the knowledge domain) given the association of the latter with attitudes.

2. A description of the instrument (domains, items, scoring methods, administration methods)

3. The development process

4. The validation process including the assessment of face validity, content validity, reliability, discriminant validity, and responsiveness.

## Results

### Search Results

**Figure 1:**
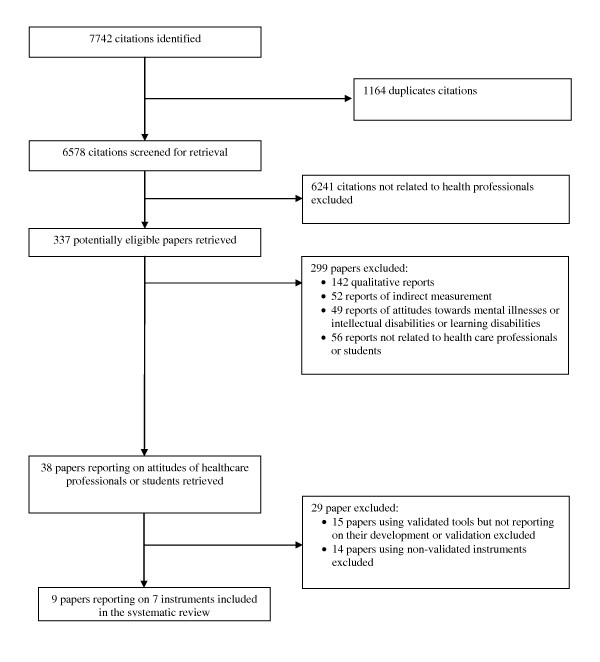
**Study Flow**.

Figure 1 describes the study flow. The screening process identified nine citations reporting on the development and/or validation of seven eligible instruments: Attitudes towards disabled people (ATDP) [25], Dental Students' Attitudes Toward the Handicapped Scale (DSATHS) [26], Scale of Attitudes Toward Disabled Persons (SADP) [27], Interaction with Disabled Persons (IDP) [28], Contact with Disabled Persons Scale (CDP) [29], Attitudes Toward Physically Disabled College Students (ATPDSC) [30,31], Rehabilitation Situations Inventory (RSI) [32]. Additional file 2 provides detailed information about the instrument (including the concept measured and the target population), its description, and its development and validation process.

We excluded 14 instruments that were not validated or were developed based on previously validated tools without additional validation (Additional file 3).

### Overview of the validated instruments

Of the seven validated instruments, four were developed for use in the adult general populations [25,27-29]. One instrument was specifically developed for dental students and dentists [26], one for rehabilitation professionals [32], and one for nursing students [30,31]. One instrument was developed in the late 1960s [25]; one was developed in the 70s [30]; three in the 80s [26,27,29]; and two in the 90s [28,32]. Six of the instruments assess attitudes [25-29,31] and one measures perceptions[30]. We included the latter instrument measuring perceptions based on a judgment that perceptions are strong determinants of attitudes.

The numbers of subscales for the different instruments are two (n = 2)[25,26], 3 (n = 3) [27,29-31], and six (n = 2) [28,32]. The number of items per instrument varies from 20 to 47. All the instruments use Likert type rating scales. Six of the instruments were designed for self-completion; this was not clear for the seventh instrument (ATPDSC). None of the studies reported completion time of the instruments.

The intended purposes of the included instruments were: evaluative (n = 3) [25,27,26], discriminative (n = 7) [25,27,28,26], predictive (n = 0), and planning (n = 1) [25].

#### Attitudes Towards Disabled People (ATDP)

ATDP measures attitudes towards disability in general and was designed for use with the general population. Of the included instruments, ATDP has been the most widely used and tested [25]. The instrument was developed in 1960. The author generated the items from literature review and discussion with psychologists. Three forms of the questionnaire are available: form O is the original form with 20 items; forms A and B, with 30 items, are improved versions of Form O. The tool has been consistently found to be reliable [5,25,33,34], and possess content and construct validity[25].

#### Dental Students' Attitudes Toward the Handicapped Scale (DSATHS)

DSATHS assesses the attitudes of dental students toward physically handicapped individuals [26]. The instrument was developed in 1983. The authors generated the items through an adaptation of previous instruments[35,36], consultations with experts, and interviews with handicapped individuals. The instrument has been found to be reliable [26], and possess content validity [26].

#### Scale of Attitudes Toward Disabled Persons (SADP)

SADP was developed to provide an alternative to the ATDP Form-O to measure the general population's attitudes towards disabilities in general [27]. The instrument was developed in 1981. It has been used to assess attitudes among occupational therapy, dental, and medical students in separate studies [27,37]. The instrument has been found to be reliable[27], and possess content validity [27].

#### Interaction With Disabled Persons (IDP)

IDP is a 20 item questionnaire that assesses attitudes in terms of level of discomfort reported by nondisabled people during interaction with people with disabilities; the type of disability was not specified [28]. The instrument was developed in 1992 and the items were generated from responses from a pool of people in response to describing how they would feel upon meeting someone with a disability, and a panel of judges assessed content validity. The instrument has been found to be reliable [28], and has been validated internationally [28].

#### Contact With Disabled Persons Scale (CDP)

While a number of items in the CDP measure the reported quantity and quality of a person's prior contact with physically disabled individuals, other items measure an affective component [29]. The responses regarding contact as well as the affective component are factored into the CDP score. The instrument was developed in 1987 and has been used in separate studies measuring attitudes among nursing, physiotherapy, and occupational therapy students. The instrument has been show to be reliable [29] and possess construct validity [29,38]. It is worth noting that the correlation of the CDP scores with those of the ATDP were marginal.

#### Attitudes Toward Physically Disabled College Students (ATPDSC)

ATPDSC assesses attitudes of nursing students toward physically disabled college students [30,31]. The questionnaire was originally developed in 1979 [30] and later modified in 1990 [31]. The instrument has been found to be reliable [30,31], and to possess face, content, and discriminant and responsiveness validity [31].

#### Rehabilitation Situations Inventory (RSI)

RSI assesses the specific behavioral situations rehabilitation professionals report as having the most difficulty in working with disabled individuals. The type of disability was not specified [32]. The instrument was developed in 1992. The authors generated the items from discussions with an expert panel of nurses, occupational and physical therapists, and psychologists. The instrument has face and content validity [39] and has been found to be reliable [32,39].

## Discussion

We identified seven validated survey instruments used for measuring attitudes of healthcare students and professionals towards patients with physical disability. Less than half were specifically designed for healthcare students and professionals and none for medical students. The most widely validated and used tool (ATDP) was developed in the late 1960s while the most recent instrument was developed in the early 1990s. We included one instrument (RSI) which measured perceptions based on a judgment that perceptions are strong determinants of attitudes.

This study has a number of strengths. This is the first systematic review of instruments validated for measuring attitudes of healthcare students and professionals towards patients with physical disability. Another strength of this study is the use of rigorous methodology, i.e. using a very sensitive and comprehensive search strategy, a duplicate and independent selection process, and a duplicate and independent data abstraction process.

The major limitation is the restriction to English language reports, leading to the possibility that relevant survey instruments in other languages were not captured in our review. However, the ATDP, SADP, and IDP were internationally validated and adapted into non-English languages [34,40-42].

The identified validated survey instruments are all at least two decades old and the majority was developed in the 1970s and 1980s. In the decades that have passed, there have been changes in societal views of people with disabilities as well as changes in legislation and public policy. As such, the identified instruments might not cover aspects relevant to today's norms or culture (e.g., using the internet, social networking to interact with disabled individuals). Furthermore some of the identified instruments used a terminology that is not relevant or socially accepted today. For example, in ATDP Form-A, people with disabilities are compared to "physically normal" people. Along the same lines, development of new instruments needs to take into account cross cultural adaptation through testing in different settings and the development of different language versions.

When choosing which of the available seven instruments to use, researchers should consider the specific research objective, the population of interest, and the unique strengths that each instrument has as detailed above. In summary, the ATDP (Form O, A, and B) has been the most widely used and tested. The DSATHS assesses the attitudes of dental students toward physically handicapped individuals. The SADP was developed to provide an alternative to the ATDP Form-O to measure the general population's attitudes towards disabilities in general. The IDP assesses attitudes in terms of level of discomfort reported by nondisabled people during interaction with people with disabilities. The CDP measures the quantity and quality of a person's prior contact with physically disabled individuals. The ATPDSC assesses attitudes of nursing students toward physically disabled college students. The RSI assesses the specific behavioral situations rehabilitation professionals report as having the most difficulty in working with disabled individuals.

## Conclusions

Medical educators need to explore the factors that affect their students' attitudes towards patients with disabilities. They also need to evaluate the impact of their educational programs to improve their students' attitudes. Using one of the identified instruments will help them in achieving valid and useful results. Obviously, a major challenge for medical educators is the limited educational time and the growing demand for education in a number of special topics such as disability, cultural competency, and sexual orientation.

Medical researchers need to develop and validate a specific instrument to measure the attitudes of medical students towards patients with physical disability. The instrument has to cover aspects relevant to today's norms and be culturally sensitive. The instrument would be helpful in exploring the factors affecting the attitudes but also the interventions aimed at improving them.

## Competing interests

The authors declare that they have no competing interests.

## Contributions

WYL and SG contributed to developing the forms, screening, data abstraction, data analysis, and drafting the manuscript. DM and IN contributed to screening. IN contributed to screening. AS contributed to drafting the protocol. EAA contributed to drafting the protocol, designing the search strategy, developing the forms, data analysis, and drafting the manuscript. All authors read and approved the final manuscript.

## Supplementary Material

Additional file 1 **Electronic search strategies**. Electronic search strategies for papers relating to attitude toward physically disabled individuals. Click here for file

Additional file 2 **Characteristics of validated survey instruments to measure attitudes of healthcare students and professionals towards patients with physical disability**. Table of the characteristics of validated survey instruments used to measure attitudes of healthcare students and professionals towards patients with physical disability. Click here for file

Additional file 3 **Non-validated survey instruments to measure attitudes of healthcare students and professionals towards patients with physical disability**. List of non-validated survey instruments that were used to measure attitudes of healthcare students and professionals towards patients with physical disability that were not included in the systematic review. Click here for file
