# Supplementary material for: Validated instruments used to measure attitudes of healthcare students and professionals towards patients with physical disability: a systematic review
Source: J Neuroeng Rehabil. 2010 Nov 9;7:55. doi: 10.1186/1743-0003-7-55 (PMC2987969; doi:10.1186/1743-0003-7-55)
Supplement: Additional file 1 — Electronic search strategies. Electronic search strategies for papers relating to attitude toward physically disabled individuals. [file 1743-0003-7-55-S1.DOCX]

**Appendix 1:** Search strategy

MEDLINE (1950-present):

| 1 | Knowledge/ or knowledge.mp. |
| --- | --- |
| 2 | Attitude/ or Attitude of Health Personnel/ or attitude$.mp. |
| 3 | belief.mp. |
| 4 | perception.mp. |
| 5 | or/1-4 |
| 6 | exp Disabled Persons/ |
| 7 | Mental Retardation/ |
| 8 | (disabled or disability).mp. |
| 9 | handicap$.mp. |
| 10 | (physical$ adj3 challenge$).mp. |
| 11 | (mental$ adj3 challenge$).mp. |
| 12 | or/6-11 |
| 13 | Data Collection/ or Questionnaires/ or Interviews as Topic/ |
| 14 | (survey$ or questionnaire$ or interview$).mp. |
| 15 | editorial.pt. |
| 16 | letter.pt. |
| 17 | Clinical Trial.pt. |
| 18 | (13 or 14) not (or/15-17) |
| 19 | 5 and 12 and 18 |

EMBASE (1980-present):

| 1 | Knowledge/ or knowledge.mp. |
| --- | --- |
| 2 | Attitude/ or Attitude to Disability/ or expHealth Personnel Attitude/ or attitude$.mp. |
| 3 | belief.mp. |
| 4 | perception.mp. |
| 5 | or/1-4 |
| 6 | Disabled Person/ |
| 7 | Mental Deficiency/ |
| 8 | (disabled or disability).mp. |
| 9 | handicap$.mp. |
| 10 | (physical$ adj3 challenge$).mp. |
| 11 | (mental$ adj3 challenge$).mp. |
| 12 | or/6-11 |
| 13 | Data Collection Method/ or Questionnaire/ or Interview/ |
| 14 | (survey$ or questionnaire$ or interview$).mp. |
| 15 | editorial.pt. |
| 16 | letter.pt. |
| 17 | (13 or 14) not (15 or16) |
| 18 | 5 and 12 and 17 |

PsycINFO (1967-present):

| 1 | Health Knowledge/ or or knowledge.mp. |  |
| --- | --- | --- |
| 2 | Attitude/ or Health Personnel Attitudes/ or “Disabled (Attitudes Toward)" / or "Mental Retardation (Attitudes Toward)"/ or "Physical Disabilities (Attitudes Toward)" or "Physical Illness (Attitudes Toward)" or attitude$.mp. |  |
| 3 | belief.mp. |  |
| 4 | perception.mp. |  |
| 5 | or/1-4 |  |
| 6 | exp Disabled Personnel/ |  |
| 7 | Mental Retardation/ |  |
| 8 | (disabled or disability).mp. |  |
| 9 | handicap$.mp. |  |
| 10 | (physical$ adj3 challenge$).mp. |  |
| 11 | (mental$ adj3 challenge$).mp. |  |
| 12 | or/6-11 |  |
| 13 | Data Collection/ or Questionnaires/ or Interviewss/ |  |
| 14 | (survey$ or questionnaire$ or interview$).mp. |  |
| 15 | (13 or 14) |  |
| 16 | 5 and 12 and 15 |  |

Health and Psychosocial Instruments (1985-present):

| 1 | knowledge.mp. |  |
| --- | --- | --- |
| 2 | attitude$.mp. |  |
| 3 | belief.mp. |  |
| 4 | perception.mp. |  |
| 5 | or/1-4 |  |
| 6 | (disabled or disability).mp. |  |
| 7 | handicap$.mp. |  |
| 8 | (physical$ adj3 challenge$).mp. |  |
| 9 | (mental$ adj3 challenge$).mp. |  |
| 10 | or/6-9 |  |
| 11 | (survey$ or questionnaire$ or interview$).mp. |  |
| 12 | 5 and 10 and 11 |  |
