# Supplementary material for: Validated instruments used to measure attitudes of healthcare students and professionals towards patients with physical disability: a systematic review
Source: J Neuroeng Rehabil. 2010 Nov 9;7:55. doi: 10.1186/1743-0003-7-55 (PMC2987969; doi:10.1186/1743-0003-7-55)
Supplement: Additional file 2 — Characteristics of validated survey instruments to measure attitudes of healthcare students and professionals towards patients with physical disability. Table of the characteristics of validated survey instruments used to measure attitudes of healthcare students and professionals towards patients with physical disability. [file 1743-0003-7-55-S2.DOC]

**Additional file 2:** Characteristics of validated survey instruments to measure attitudes of healthcare students and professionals towards patients with physical disability

| **Instrument** | **Description of the instrument** | **Development** | **Validation** | **Example items** |
| --- | --- | --- | --- | --- |
| - Attitudes towards disabled people (ATDP) (Yuker 1970) - Concept: attitudes toward people with disability in general - Target population: general population | - Three forms: O (original; 20 items), A and B (2 attempts at improving form O; 30 items) - Domains:   - Characteristics   - Treatment - Scoring method: 6 point Likert scale; -3 (I disagree very much) to +3 ( I agree very much) - Administration method: self-administered | - Identification of items: literature review - Initial pool of 300 items - Selection of items: psychologists (relevance) - Revised pool of items after screening for face validity and modifying the wording: 40-60 items - Final version after internal item analysis (screen the discriminative ability of each item): 20 items (Form O); 30 items (Forms A, B) | - Test-retest reliability: correlation coefficient: (0.66 – 0.89) (Yuker 1970), (0.67-0.70) (Yuker and Block 1986)(form O); (0.78) (Yuker 1970), (0.74-0.91) (Yuker and Block 1986)(Form A); (0.71 – 0.83) (Yuker 1970), (0.83-0.85) (Yuker and Block 1986) (Form B) - Internal consistency: cronbach’s alpha (0.79) (Tervo, Palmer et al. 2004) (0.76) (Yuker and Block 1986) (Form O); (0.78) (Chan, Lee et al. 2002), (0.86) (Chan, Lee et al. 2002), (0.90)(Lee, Paterson et al. 1994), and (0.83-0.85) (Yuker and Block 1986)(Form A); (0.79-0.89) (Yuker and Block 1986)(Form B) - Split-half equivalence reliability: coefficient (Yuker 1970) (0.75-0.85) (Yuker 1970; Yuker and Block 1986)(Form O); (0.73-0.89) (Yuker 1970), (0.72-0.91) (Yuker and Block 1986)(Form A); (0.72-0.87) (Yuker 1970; Yuker and Block 1986)(Form B) - Content validity (comprehensive literature review and item analysis) (Forms A, B, O) (Yuker 1970) - Criterion-related and construct validity (correlating ATDP scores with personality characteristics, behavior, self-concept, and prejudice) (Forms A, B, O) (Yuker 1970) | Disabled people are often unfriendly; disabled people should have to compete for jobs with physically normal persons. |
| - Name: Dental Students’ Attitudes Toward the Handicapped Scale (DSATHS)(Lee, Sonis et al. 1983) - Concept: attitudes toward people with physical disability - Target population:   dental students | - Domains (32 items):   - Effect of instructors’ experiential qualifications on attitudes toward working with the handicapped (19)   - Desire for future and interpersonal relationships with the handicapped (13) - Scoring method: 5 point Likert scale; 1 (strongly disagree) to 5 (strongly agree) - Administration method: self-administered | - Identification of items:   - Experts   - Adaptation of previous instruments (Yuker 1960; McTigue 1978)   - Personal interviews with handicapped individuals   - Initial pool of 178 items - Selection of items:   - Experts (checked face validity, grammar)   - Revised pool of 108 items - Final pool of 32 items after validity assessment | - Internal consistency: correlational analysis: r=0.756 (p<0.05) (Lee, Sonis et al. 1983) - Test-retest reliability: 2 administrations of the instrument to 38 freshmen dental students (Pearson r=0.844; p<0.001) (Lee, Sonis et al. 1983) - Content validity established by 1) factor analysis and simplified by varimax rotation; 2) item analysis; and 3) correlation studies (Lee, Sonis et al. 1983) | I am not interested in learning anything else about handicapped people; in the private office, a separate waiting room should be provided for disfigured patients. |
| - Name: Scale of Attitudes Toward Disabled Persons (SADP) (Antonak 1981) - Concept: attitudes toward people with disability in general - Target population: general population | - Domains (24 items):   - Optimism/Human Rights   - Behavioral Misconceptions   - Pessimism/Hopelessness - Scoring method: 144 point Likert scale; 0 (very negative) to 144 (very positive) - Administration method: self-administered | - Identification of items: not reported - Selection of items: not reported | - Test-retest reliability: Spearman-Brown corrected coefficient (0.81-0.85) (Antonak 1988) - Spearman-Brown corrected coefficient for subscales: 0.71, 0.55, and 0.61, respectively (Antonak 1988) - Internal consistency: Cronbach’s alpha (0.88-0.91) (Antonak 1988) - Cronbach’s alpha coefficients for the subscales are 0.81, 0.77, and 0.82, respectively (Antonak 1981) - Reported as having content validity (Antonak 1981) | Disabled children should be provided with a free public education; disabled people should be prevented from having children |
| - Name: Interaction with Disabled Persons (IDP) (Gething and Gething 1992) - Concept: attitudes toward people with disability in general - Target population: general population | - Domains (20 items with 3 items loading on to 6 factors):   - Discomfort in social interaction (6)   - Coping/succumbing framework (4)   - Perceived level of information (5)   - Vulnerability (2)   - Coping (2)   - Vulnerability (2) - Scoring method: 6 point scale - Administration method: self-administered | - Identification of items: content analysis of responses from 633 people - Initial pool of 30 items - Selection of items: panel of judges assessed face and content validity. - Final pool of 20 items after psychometric evaluations to enhance construct validity | - Test-retest reliability: coefficient (0.51-0.82) (Gething and Gething 1992) - Internal consistency: Cronbach’s alpha (0.74-0.86) (Gething and Gething 1992) - International validation: Cronbach’s alpha coefficients (moderate to high range) indicating that IDP scale is a sensitive measure to discriminate among respondents in 9 countries (Gething and Gething 1992) | I feel overwhelmed with discomfort about my lack of disability; I am afraid to look at the person straight in the face |
| - Name: Contact with Disabled Persons Scale (CDP) (Yuker 1987) - Concept: attitudes toward people with physical disability - Target population: general population | - Domains (20 items):   - Amount of prior contact   - Type of prior contact   - Affective component - Scoring method: 5 point Likert scale; 1 (never) to 5 (very often); total score range of 20-100 - Administration method: self-administered | - Identification of items: not reported - Selection of items: not reported | - Test-retest reliability: median coefficient (corrected estimates) (0.93) (Yuker 1987) - Median coefficient (alpha estimates) (0.92) (Yuker 1987) - Median split half reliability (0.87) (Yuker 1987) - Construct validity:   - Correlation of CDP scores with attitude scores on ATDP scale: (r=-0.26 to 0.40) (Yuker 1987)   - Correlation of CDP scores with variables: years of rehabilitation practice (0.29); years of full-time nursing practice (0.21) (Geskie 1985) | How often have you met a disabled person for whom you feel sorry? How often have you met a physically disabled person that you like? |
| - Name: Attitudes Toward Physically Disabled College Students (ATPDSC) (Rice 1979; Messmer 1990) - Concept: attitudes toward people with physical disability - Target population: nursing students | - Domain (47 items):   - Attitudes- in class experience (22)   - Attitudes- out of class experience (7)   - Attitudes- mainstreaming disabled into collegiate setting (18) - Scoring method: 5 point Likert scale; strongly agree to strongly disagree - Administration method: not reported | - Originally developed by Rice (1979) (Rice 1979):   - Identification of items: panel of experts   - Selection of items: pretested items on group of college students - Modified by Messmer (Messmer 1990):   - Identification of items: Adaptation of previous instrument (Rice 1979)   - Selection of items: panel of experts from nursing and rehabilitation counseling to reaffirm content validity | - Test-retest reliability: coefficient (0.75) (Rice 1979) - Item reliability: Cronbach’s alpha 0.78 (pretest), 0.80 (posttest) (Messmer 1990) - Face validity (reaffirmed by researcher); - Content validity (panel of experts); - Discriminant validity (contact vs. no contact with disabled persons) (Messmer 1990): - Responsiveness (before and after a course) (Messmer 1990) | In any situation it is all right for non-disabled students to be seen socially with disabled students on the same campus; most disabled college students are more dependable than non-disabled students in carrying out what they promised to do in the academic classroom |
| - Name: Rehabilitation Situations Inventory (RSI) (Dunn ME 1992; Dunn 1996) - Concept: perception toward people with physical disability - Target population: rehabilitation professionals | - Domain (30 items):   - Aggression (6)   - Sexual Situations (4)   - Staff (4)   - Families (5)   - Depression (3)   - Motivation/ Adherence (7) - Scoring method: Likert-type, scale of 1-5 - Administration method: self administered | - Identification of items: expert panel of nurses, occupational and physical therapists, and psychologists (discussions) - Initial pool of 95 items - Selection of items: preliminary tool administered to 177 rehabilitation staffs - Final inventory of 30 items selected by examining the medians and inter-quartile ranges of the 95 items - Subscale development:   - Visual inspection of items (6 face-valid categories) (k=0.83) (Dunn 1996)   - Factor analysis (6 subscales) (inter-subscale correlations 0.44-0.68) (Dunn 1996) | - Test-retest reliability, correlation:   - 0.73-0.87 for subscales (Dunn 1996) - Internal consistency, Pearson’s coefficient:   - 0.88 for instrument (Dunn ME 1992) - Internal consistency, Cronbach’s alpha:   - 0.93 for instrument (Dunn ME 1992)   - 0.72-0.84 for subscales (Dunn 1996) | Some situations that staff encounter in a rehabilitation setting are difficult to handle, awkward, or embarrassing. Please evaluate how difficult you would find each of the following, i.e. patient says “I just feel like giving up” or “I’m old. I’d rather be dead than injured.” |

**References:**

Antonak, R. F. (1981). "Development and psychometric analysis of the Scale of Attitudes Toward Disabled Persons. (Tech. Rep. No. 5)."

Antonak, R. F., Livneh, H (1988). The measurement of attitudes toward people with disabilities: methods, psychometrics and scales. Springfield IL, Charles C Thomas.

Chan, C. C. H., T. M. C. Lee, et al. (2002). "Attitudes toward people with disabilities between Chinese rehabilitation and business students: An implication for practice." Rehabilitation Psychology **47**(3): 324-338.

Dunn, M. (1996). "Subscale development of the rehabilitation situations inventory." Rehabil Psychol **41**: 255-264.

Dunn ME, U. R., Mermis BJ (1992). "The rehabilitation situations inventory; staff perception of difficult behavioral situations in rehabilitation." Arch Phys Med Rhabil **73**: 316-319.

Geskie, M. A. (1985). "The relationship between empathy, attitudes toward disabled persons, and level of nursing education." Dissertation Abstracts International **47**(850B).

Gething, L. and L. Gething (1992). "Nurse practitioners' and students' attitudes towards people with disabilities." Australian Journal of Advanced Nursing **9**(3): 25-30.

Lee, M. M., A. L. Sonis, et al. (1983). "An instrument to assess dental students' attitudes toward the handicapped." Special Care in Dentistry **3**(3): 117-123.

Lee, T. M., J. G. Paterson, et al. (1994). "The effect of occupational therapy education on students' perceived attitudes toward persons with disabilities." American Journal of Occupational Therapy **48**(7): 633-638.

McTigue, D., Musselman, R, and Rasmussen, R (1978). Validation of a needs assessment instrument in developing a curriculum for dentistry for the special patient. Paper presented at the annual meeting of the American Association of Dental Schools. Washington, DC.

Messmer, P. R. (1990). Nursing students' attitudes toward physically disabled college students. Measurement of nursing outcomes. C. F. Waltz and O. L. Strickland. New York, Springer. **3:** 203-219.

Rice, D. (1979). An investigation of the attitudes of the able-bodied college student toward the physically handicapped college student in the competitive academic setting, University of Pittsburgh.

Tervo, R. C., G. Palmer, et al. (2004). "Health professional student attitudes towards people with disability." Clinical Rehabilitation **18**(8): 908-915.

Yuker, H. E. and J. R. Block (1986). Research with the attitude toward disabled persons scale (ATDP). Hempstead (NY), Center for the study of Attitudes towards persons with disability. Hofstra University.

Yuker, H. E., Block, J. R., & Campbell, W (1960). A scale to measure attitudes disabled persons (no. 3). Albertson, NY, Human Resources Foundation.

Yuker, H. E., Block, J. R., & Younng, J. H. (1970). Measurement of attitudes toward disabled persons. Albertson, NY, INA Men Institute at Human Resources Center.

Yuker, H. E., Hurley, M. K. (1987). "Contact with and attitudes toward persons with disabilities: The Measurement of Intergroup Contact." Rehabilitation Psychology **32**(3): 145-154.
