# Supplementary material for: Validated instruments used to measure attitudes of healthcare students and professionals towards patients with physical disability: a systematic review
Source: J Neuroeng Rehabil. 2010 Nov 9;7:55. doi: 10.1186/1743-0003-7-55 (PMC2987969; doi:10.1186/1743-0003-7-55)
Supplement: Additional file 3 — Non-validated survey instruments to measure attitudes of healthcare students and professionals towards patients with physical disability. List of non-validated survey instruments that were used to measure attitudes of healthcare students and professionals towards patients with physical disability that were not included in the systematic review. [file 1743-0003-7-55-S3.DOCX]

**Additional file 3:** Non-validated survey instruments to measure attitudes of healthcare students and professionals towards patients with physical disability

| **Instrument** | **Description of the instrument** | **Development** |
| --- | --- | --- |
| - Eberhardt 1995 (Eberhardt, Mayberry et al. 1995) - Name: Disability Social Distance Scale (DSDS) - Concept: attitudes | - Domains (9 items):   - Social distance (9) - Scoring method: rank order - Administration method: self administered | - Modification of the Disability Social distance scale (DSDS) (Deletion of several presently uncommon disabilities (e.g., tuberculosis) and addition of more current types (e.g., AIDS); modification of 2 categories of social distance (e.g., best friend instead of next-door neighbor); addition of 4 new situations to the 27 disabilities on the scale) |
| - Cooper 2003 (Cooper, Rose et al. 2003) - Name: Attitudes towards deaf people scale - Concept: attitudes | - Domains (22 items): - equality - ability - cultural issues - linguistic issues - Scoring method: 6 point Likert scale; yes/no options - Administration method: self administered | - Identification of items: focus group interview - Selection of items: experts (relevance); data driven (item analysis) |
| - Eberhardt 1994 (Eberhardt 1994) - Name: Similarity Scale - Concept: perceptions | - Total items: 7 - Scoring method: Likert scale (5-point)   Administration method: self administered | - Developed by authors to explore equal status contact - Development and validation details not reported |
| - Aulagnier 2005 (Aulagnier, Verger et al. 2005) - Concept: knowledge, attitudes | - Domain (13 items):   - Factors Potentially Associated with Knowledge (4)   - Factors Affecting Attitudes Towards Persons with Disabilities (2)   - External Factors Affecting Working Conditions (6)   - Specific Practices in caring for Patients With Disabilities (1) - Scoring method: not reported - Administration method: researcher-administered | - Development through consultation with 3 GPs who had worked with peer groups for discussions about GPs’ practices in the field; questionnaire was constructed by a research team that has participated in the design of “Knowledge, Attitudes, Belief & Practice” studies in several fields |
| - Westbrook 1988 (Westbrook, Adamson et al. 1988) - Concept: knowledge, perceptions | - Total items: 18 - Scoring method: not reported - Administration method: interviewer-administered | - Not reported |
| - McKenna 2001 (McKenna, Scholtes et al. 2001) - Concept: attitudes, perceptions | - Domain (10 items):   - Attitudes to disability   - Perceptions of the characteristics of a successful occupational therapist   - Reasons underlying decision to enter occupational therapy course   - Expectations of the occupational therapy course   - Career Plans - Scoring method: Likert-type scale; 4 point (from very important to not at all important) - Administration method: self-administered | - Developed by Fleming, 1997 (Fleming, Gilbert et al. 1997) |
| - Rose 1999 (Rose 1999) - Concept: beliefs, attitudes | - Total items: not reported - Scoring method: not reported - Administration method: self-administered | - Development: written survey developed specifically for the study addressing accessibility of healthcare and suggestions from chiropractic college clinicians who were pilot tested |
| - Singer 1983 (Singer P 1983) - Concept: attitude | - Total items: not reported - Scoring method: not reported - Administration method: self-administered | - Not reported |
| - Dolan 1983 (Dolan, Sawyer et al. 1983) - Concept: attitudes, beliefs | - Domain:   - "mobility of blind persons"   - "persons who are blind"   - "deaf persons"   - "my thoughts about handicapped people,"   - "communicating with handicapped people" - Total items: not reported - Scoring method: 7 point rating scale - Administration method: self-administered | - Not reported |
| - Westbrook 1988 (Westbrook, Adamson et al. 1988) - Concept: knowledge | - Domain (30 items):   - Prevalence of handicaps (17)   - Knowledge of Sex Differences in Handicaps (13) - Scoring method: not reported - Administration method: self-administered | - Not reported |
| - Molnar 1987 (Molnar and Knasel 1987) - Concept: knowledge and attitudes | - Total items: 12 - Scoring method: 5-point scale (from strongly disagree to strongly agree) - Administration method: self-administered | - Developed by the authors; details not reported - Internal consistency and test-retest reliability: (0.49-0.65) moderate to moderately high coefficients |
| - Miller 1976 (Miller and Heil 1976) - Concept: attitudes | - Total items: 20 - Scoring method: 7-point scale (from strongly disagree to strongly agree) - Administration method: self-administered | - Developed by the authors to measure attitudinal change as an effect of extramural program of dental care for the special patient on attitudes of dental students; no further details reported |
| - Watson 1979 (Watson, Brundo et al. 1979) - Concept: attitudes | - Total items: not reported - Scoring method: not reported - Administration method: self-administered | - Developed by the authors; details not reported |
| - Mitchell 1991 (1991) - Concept: attitudes | - Total items: 20 - Scoring method: 7-point scale (from strongly disagree to strongly agree) - Administration method: self-administered | - Developed by the authors to measure attitudinal change as an effect of extramural program of dental care for the special patient on attitudes of dental students; no further details reported |

**References**

Aulagnier, M., P. Verger, et al. (2005). "General practitioners' attitudes towards patients with disabilities: The need for training and support." Disability and Rehabilitation **27**(22): 1343-1352.

Cooper, A. E., J. Rose, et al. (2003). "Mental health professionals' attitudes towards people who are deaf." Journal of Community & Applied Social Psychology **13**(4): 314-319.

Dolan, J., H. W. Sawyer, et al. (1983). "Presence versus absence of others: The effect on verbally expressed attitudes toward the disabled." Rehabilitation Psychology **28**(4): 239-242.

Eberhardt, K., W. Mayberry, et al. (1995). "Factors influencing entry-level occupational therapists' attitudes toward persons with disabilities." American Journal of Occupational Therapy **49**(7): 629-636.

Eberhardt, K. E., & Mayberry, W. (1994). Similarity Scale.

Fleming, J., J. Gilbert, et al. (1997). "First year occupational therapy students: Profile and perceptions." Australian Occupational Therapy Journal **44**(3): 107-118.

McKenna, K., A. A. Scholtes, et al. (2001). "The journey through an undergraduate occupational therapy course: Does it change students' attitudes, perceptions and career plans?" Australian Occupational Therapy Journal **48**(4): 157-169.

Miller, S. and J. Heil (1976). "Effect of an extramural program of dental care for the special patient on attitudes of students." J Dent Ed **40**(11): 740-744.

Mitchell, J. (1991). "Attitudes of and toward older persons with a disability: Their measurement and their role in rehabilitation. ." Journal of Rehabilitation Research and Development **28**(1): 140-141.

Molnar, E. T. and A. L. Knasel (1987). "Evaluation of pediatric community field trips." Journal of the National Medical Association **79**(5): 513-517.

Rose, K. A. (1999). "A survey of the accessibility of chiropractic clinics to the disabled." Journal of Manipulative and Physiological Therapeutics **22**(8): 523-529.

Singer P, K. H., Singer C (1983). "The treatment of newborn infants with major handicaps. A survey of obstetricians and paediatricians in Victoria." Medical Journal of Australia **2**: 274-278.

Watson, J. F., G. C. Brundo, et al. (1979). "Attitudinal differences of faculty and students regarding the care of special handicapped patients in a dental school clinic." Journal of the American Dental Association **98**(3): 395-397.

Westbrook, M. T., B. J. Adamson, et al. (1988). "Health science students' images of disabled people." Community Health Studies **12**(3): 304-313.
